# The pervasive impact of global climate change on plant-nematode interaction continuum

**DOI:** 10.3389/fpls.2023.1143889

**Published:** 2023-04-06

**Authors:** Tushar K. Dutta, Victor Phani

**Affiliations:** ^1^Division of Nematology, ICAR-Indian Agricultural Research Institute, New Delhi, India; ^2^Department of Agricultural Entomology, College of Agriculture, Uttar Banga Krishi Viswavidyalaya, West Bengal, India

**Keywords:** plant-parasitic nematodes, geographic distribution, global climate change, population dynamics, elevated CO2 level, altered precipitation, sustainable management, agroecosystem

## Abstract

Pest profiles in today’s global food production system are continually affected by climate change and extreme weather. Under varying climatic conditions, plant-parasitic nematodes (PPNs) cause substantial economic damage to a wide variety of agricultural and horticultural commodities. In parallel, their herbivory also accredit to diverse ecosystem services such as nutrient cycling, allocation and turnover of plant biomass, shaping of vegetation community, and alteration of rhizospheric microorganism consortium by modifying the root exudation pattern. Thus PPNs, together with the vast majority of free-living nematodes, act as ecological drivers. Because of direct exposure to the open environment, PPN biology and physiology are largely governed by environmental factors including temperature, precipitation, humidity, atmospheric and soil carbon dioxide level, and weather extremes. The negative effects of climate change such as global warming, elevated CO_2_, altered precipitation and the weather extremes including heat waves, droughts, floods, wildfires and storms greatly influence the biogeographic range, distribution, abundance, survival, fitness, reproduction, and parasitic potential of the PPNs. Changes in these biological and ecological parameters associated to the PPNs exert huge impact on agriculture. Yet, depending on how adaptable the species are according to their geo-spatial distribution, the consequences of climate change include both positive and negative effects on the PPN communities. While assorting the effects of climate change as a whole, it can be estimated that the changing environmental factors, on one hand, will aggravate the PPN damage by aiding to abundance, distribution, reproduction, generation, plant growth and reduced plant defense, but the phenomena like sex reversal, entering cryptobiosis, and reduced survival should act in counter direction. This seemingly creates a contraposition effect, where assessing any confluent trend is difficult. However, as the climate change effects will differ according to space and time it is apprehensible that the PPNs will react and adapt according to their location and species specificity. Nevertheless, the bio-ecological shifts in the PPNs will necessitate tweaking their management practices from the agri-horticultural perspective. In this regard, we must aim for a ‘climate-smart’ package that will take care of the food production, pest prevention and environment protection. Integrated nematode management involving precise monitoring and modeling-based studies of population dynamics in relation to climatic fluctuations with escalated reliance on biocontrol, host resistance, and other safer approaches like crop rotation, crop scheduling, cover cropping, biofumigation, use of farmyard manure (FYM) would surely prove to be viable options. Although the novel nematicidal molecules are target-specific and relatively less harmful to the environment, their application should not be promoted following the global aim to reduce pesticide usage in future agriculture. Thus, having a reliable risk assessment with scenario planning, the adaptive management strategies must be designed to cope with the impending situation and satisfy the farmers’ need.

## 1 Introduction

Climate change is perceived by the durable shift in Earth’s weather that occurs naturally and by anthropogenic causes. The most pervasive consequence of this phenomenon is reflected through global warming. According to the UNEP Emissions Gap Report (2022), the current track for temperature rise may reach up to 2.4-2.6°C increase by the end of this century (www.unep.org/resources/emissions-gap-report-2022). Though the extensive amount of greenhouse gases (GHGs; methane, carbon dioxide and nitrous oxide) emitted by fossil-fuel burning and change in land-use pattern primarily drive the temperature increase, natural factors like solar luminosity, radiation budget, volcanic eruptions, and Earth’s orbital variations round the sun also act as contributing factors. Simultaneously, the changes in precipitation pattern and extreme weather events are likely to intensify (www.epa.gov/climate-indicators). The permanent and violent alterations in these abiotic factors eventually affect the Earth’s biome including wildlife, biological extinctions, ecosystems and agriculture. With the rising population at present, the global food production needs to be doubled by 2050 (Tilman et al., 2011). Several recommendations were made to maintain the food security in a sustainable way with concomitant protection of the environment. In the upcoming time, climate change is going to hit agriculture by bringing uncertainties in food production (Tirado et al., 2013), and the effects are conceived in both positive and negative directions for the abiotic and biotic drivers of the agroecosystem.

The changes in climatic factors, as reflected by rising atmospheric temperature, elevated level of carbon dioxide, altered precipitation, and increased proportion of natural extremes such as heat wave, flood and drought are likely to affect the tripartite interactions between the hosts, parasites and environment. The interacting-trio acts simultaneously at each levels of spatio-temporal scale to manifest the disease (Duffy et al., 2012; Kalinkat et al., 2015). Being the parts of same ecosystem, the host and the parasite depend on the ecological settings and vary as well with the changes (Lazzaro and Little, 2009; Wolinska and King, 2009). An individual host, with which a parasite interacts, may act as the parasite’s fostering ecosystem (Rynkiewicz et al., 2015), but the spatial distribution of host infection depends on the environment (Penczykowski et al., 2016). In case of tight dependency of the parasites on their hosts, environmental changes can affect both the associates in an asymmetrical way. The hosts, if widely distributed and persistent over evolutionary time scale, favor more specialist parasites to evolve, although they are less prone to extinction (Strona, 2015). Conversely, for the parasites, strict host specificity offers fewer choices in an unstable host population (Cizauskas et al., 2017). As a result, the specialist parasites face much deleterious effects than the generalists. Thus, the distribution, reproduction, population dynamics and survival of a parasite is determined by a complicated nexus of host factors (e.g. adaptability, density, transmission, physiological tolerance) and parasitic factors (e.g. specificity, dependency, complexity in life cycle), both of which are independently affected in the changing environment (Martinez and Merino, 2011; Skendžic et al., 2021). For obligate dependency of the parasites, climatic shifts aggravate their decline in population (Singer et al., 2013). Further, the external environmental changes directly affect the parasites having free-living stages in their life cycle (Mas-Coma et al., 2008; Froeschke et al., 2010; Okulewicz, 2017). Plant-parasitic nematodes (PPNs) though do not contain a ‘true’ free-living stage but the presence of non-feeding soil-dwelling infective stages indicates that PPNs are not exempted from the dire consequences of climate change. Thus, the patterned shift in ecology and pest biology will obviously pose an alarming threat to the crop production system. Therefore, finding a seminal solution for the problems associated with PPNs, which are obligate parasites with both specific and general host preferences, is problematic.

## 2 Nematodes as ecosystem drivers in the climate change continuum

Nematodes form an integral part of soil biodiversity having direct or indirect influence on the ecosystem processes. In particular, the higher latitudes are more dominated by the nematodes and other micro-arthropods (Ruess et al., 1999). Broadly, the soil-inhabiting nematode fauna are classified as free-living and parasitic (to both plants and animals). The feeding of free-living nematodes on the soil bacteria, fungi and other nematodes contribute to litter decomposition, carbon and nitrogen mineralization, alteration of C:N ratio, nutrient recycling and carbon sequestration (Ferris and Bongers, 2006; Neher, 2010; Nielsen et al., 2014). They also help in soil microbe distribution regulating their turnover in soils. Notably, nematode biomass (0.3 gigatonnes) in the global topsoil corresponds to approximately 80% of collective human biomass on planet Earth. Besides, the quantity of carbon respired (0.11 gigatonnes C per month) by the soil inhabiting nematodes is equivalent to approximately 15% of carbon emissions from fossil fuel use (van den Hoogen et al., 2019).

Alternatively, majority of the PPNs thrive by subsurface herbivory (some are above-ground parasites including *Aphelenchoides* spp., *Bursaphelenchus* spp., *Anguina* spp.) and cause damage to the crops. Around 4100 species of PPNs exert an estimated yearly loss of US$ 173 billion to the agricultural and horticultural crops under open-field and protected conditions (Elling, 2013; Jones et al., 2013; Phani et al., 2021). The economically important and widely distributed common PPN genera include root-knot (*Meloidogyne* spp.), cyst (*Heterodera* spp., *Globodera* spp.), lesion (*Pratylenchus* spp.), burrowing (*Radopholus* spp.), reniform (*Rotylenchulus* spp.), false root-knot (*Nacobbus* spp.), stem and bulb (*Ditylenchus* spp.), foliar (*Aphelenchoides* spp.) nematodes; and the ectoparasites like dagger (*Xiphinema* spp.), needle (*Longidorus* spp.), stubby-root (*Trichodorus* spp.), ring (the criconematids), awl (*Belonolaimus* spp.), sting (*Belonolaimus* spp.), spiral (*Helicotylenchus* spp.), stunt (*Tylenchorhynchus* spp.) and lance (*Hoplolaimus* spp.) nematodes. They either induce specialized feeding cells on plant roots (e.g. giant cells by root-knot nematodes, syncytia by cyst nematodes, nurse cells by reniform nematode), or feed from inside or outside the roots affecting nutrient availability to the plants. Thus the PPNs remarkably maneuver the physiology, root architecture, nutrient uptake and water use efficiency of plants by developing intimate (for semiendo- and endoparasites) or non-intimate (for ectoparasites) host-pathogen relationship (Eves-van den Akker, 2021; Siddique et al., 2022). In addition, many PPN species predispose the infected plants to fungal, bacterial and viral pathogens that synergize the damage toll (Khan, 1993). Thus, the PPNs are often marked with a ‘pathogen badge’ from an agroecological perspective. But, in a broader ecological aspect, their plant feeding habit considerably drives the plant performance under natural ecosystems by plant-soil feedbacks (Wilschut and Geisen, 2021). Greater amount of nematode herbivory reduces the plant biomass in general, and changes the biomass allocation pattern as seen in grasslands (Brinkman et al., 2015; Franco et al., 2020). The nematodes also help to shape the vegetation community aboveground (Hedenec et al., 2022). As seen in the coastal dune grasslands, the specialist PPNs play pivotal role in natural succession by bringing the end of dominant plant species followed by the non-host plant establishment (van der Putten et al., 1993). The generalist root feeding nematodes, while accumulated in higher numbers in the rhizosphere of flourishing annuals, possibly contribute in turnover from ephemeral to stable grass species. Although the variation in generalist accumulation is yet to be conclusively correlated with the variation of plant-soil feedback (Wilschut et al., 2019), nematode herbivory evidently contributes to the plant community dynamics. Further, the PPNs can alter the plant performance by changing the root exudation pattern that helps to modify the rhizospheric microbial population and enhance N and P availability to plants (Neher, 2001; Tu et al., 2003; Topalovic et al., 2020). In this line, Verschoor (2002) found that plant feeding by nematodes aid in nutrient cycling by excretion of ammonia, N defecation, and increment in root exudation. The herbivory may also contribute to soil C increase, as observed by measurable ^14^C increase in soil upon nematode infection in ^14^C labeled plants (Yeates et al., 1993).

The diversity, abundance and pathogenic potential of the PPNs are largely shaped by a complex union of various factors such as plant hosts, regulation by natural enemies, inter- and intraspecific competitions, and climatic factors. Amongst the abiotic factors, temperature, humidity and precipitation pattern potentially influence the survival, development and behavior of the soil-inhabiting PPNs (Norton, 1979). Alike other parasites and pathogens, the warm and humid climate favors quick development, higher reproduction, increased period of transmission, altered sex ratio, spatiotemporal shift in abundance and diversity of both the plant-parasitic and free-living nematodes (Neilson and Boag, 1996; Yeates and Newton, 2009; Maleita et al., 2012; Wu et al., 2016; St-Marseille et al., 2019; Guo et al., 2021; Klusmann et al., 2022). The micro-parasitic abundance in an agroecosystem is directly linked to the degree of host damage and economic loss of the crops. Individual parasite, by their own accord (if not a vector) inflicts minor damage that plants can withstand (Irvin et al., 2006), although climate change and global warming seem to favor higher development of the poikilothermic parasites (Culos and Tyson, 2014), as apprehensible for the PPNs. Moreover, warmer temperature changes the geographical distribution pattern of the nematodes. This creates the major problem of pest invasion into new areas, and also helps to grow minor pests as major ones for range expansion of the species. As the nematode trophic groups are interconnected *via* complex ecological network of plant, soil and environment, understanding the impact of different functional groups on crop production under changing global climate is necessary to achieve sustainability in crop production. Among the multitude of climate change factors, increase in atmospheric CO_2_, temperature variability, alteration of precipitation level and pattern, and climatic extremes maybe contemplated as the dominant drivers for shifting community composition of the PPNs (Colagiero and Ciancio, 2012; Kandel et al., 2013; Mueller et al., 2016). In the following paragraphs we will discuss the consequences of global climate change on the plant feeding nematodes.

## 3 Effect of global climate change on plant-parasitic nematodes

The responses of different plant feeding nematodes towards various climate change parameters like increased temperature, elevated CO_2_ and altered precipitation level are compiled in **Table 1**.

**Table 1:** Variable response of plant-parasitic nematodes to altered temperature, CO_2_ and precipitation level.

Ecosystem/Cropping system	Experimental system	Nematode species	Nematode abundance	Country	Reference
Effect of increased temperature					
Pine forest	Ecosystem monitoring, tree sampling	*Bursaphelenchus mucronatus*, *B. xylophilus*	Increased prevalence	Switzerland	Rebetez and Dobbertin, 2004
Coffee plantation	Simulated model	*Meloidogyne incognita*	Increased pathogenic potential	Brazil	Ghini et al., 2008
Carrot	Simulated model	*Heterodera carotae*	Increased juvenile and egg production	Italy	Colagiero and Ciancio, 2012
Grassland	Light scaffolding in field soil by using curtains of high-density polyethylene mesh	*Paratylenchus* spp., *Filenchus* spp., *Helicotylenchus* spp.	Increase in herbivorous nematodes with extended life span	Denmark	Stevnbak et al., 2012
Grassland	Fields were warmed by infrared radiators	*Aphelenchoides* spp., *Paratylenchus* spp., *Pratylenchus* spp, *Rotylenchus* spp.	Daytime warming (compared to night-time and diurnal warming) had stronger negative effect on nematode abundance	China	Yan et al., 2017
Potato	Controlled environment, simulated model	*Globodera rostochiensis*, *G. pallida*	Increased abundance of *G. rostochiensis* but not *G. pallida*	United Kingdom	Jones et al., 2017
Rice-wheat rotation	Infrared heating lamps in open field for canopy warming	*Filenchus* spp.*, Psilenchus* spp.*, Hirschmanniella* spp.*, Pratylenchus* spp.	Not affected (although nematode diversity increased)	China	Wang et al., 2021
Tomato	Controlled environment	*Meloidogyne incognita*	Nematode population growth was significantly affected despite increase in plant root and shoot growth	India	Berliner et al., 2023
Effect of CO_2_ enrichment					
Ryegrass/white clover	Controlled environment	*Dorylaimus* spp., *Trichodorus* spp.	Increased	New Zealand	Yeates et al., 1997
Pasture or grassland	Natural vent	*Pratylenchus* spp.,	Increased	New Zealand	Yeates et al., 1999
Grassland	Open top chambers	*Meloidogyne* spp., *Neopsilenchus* spp., *Paratylenchus* spp., *Gracilacus* spp., *Xiphinema* spp.	Increased	USA	Hungate et al., 2000
Grassland	Free air CO_2_ enrichment	*Tylenchus* spp., *Longidorus* spp.	Increased	New Zealand	Yeates et al., 2003
Grassland	Screen-aided CO_2_ control	Species name not provided	Not affected	Switzerland	Niklaus et al., 2003
Grassland	Free air CO_2_ enrichment	Species name not provided	Increased earlier, later returned to baseline level	Germany	Sonnemann and Wolters, 2005
Rice, wheat rotation	Free air CO_2_ enrichment	*Psilenchus* spp., *Hirschmanniella* spp., *Filenchus* spp., *Tylenchus* spp.	Increased	China	Li et al., 2007; Li et al., 2009
Grassland	Open top chambers	*Heterodera* spp., *Meloidogyne* spp., *Longidorus* spp., *Trichodorus* spp., *Criconema* spp., *Tylenchus* spp., *Tylenchulus* spp.	Not affected despite increase of Anguinid root feeders	USA	Ayres et al., 2008
Grassland	Controlled environment	Do	Not affected	France	Ayres et al., 2008
Sugar beet, wheat rotation	Free air CO_2_ enrichment	Species name not provided	Increased	Germany	Sticht et al., 2009
Rice	Open top chambers	*Meloidogyne graminicola*	Not affected	India	Somasekhar and Prasad, 2012
Rice-wheat rotation	Free air CO_2_ enrichment	*Filenchus, Psilenchus, Hirschmanniella, Pratylenchus*	Not affected (although nematode diversity increased)	China	Wang et al., 2021
Effect of altered precipitation					
Grassland, cereals	Field sampling	*Meloidogyne minor*, *Heterodera* spp. and *Pratylenchus* spp.	Increased	Northern Ireland	Fleming et al., 2016
Arid, semiarid and mesic grasslands	Precipitation manipulation by using rainout shelters in field plots	Species name not provided	Reduced precipitation in mesic grassland increased plant feeder abundance	USA	Franco et al., 2019
Blue grama grass, *Bouteloua gracilis*	Controlled environment in greenhouse	Species name not provided	Reduced precipitation in sub-humid grassland increased root herbivory	USA	Franco et al., 2020
Desert grassland, semiarid shortgrass steppe, mesic tallgrass prairie	Precipitation manipulation by using rainout shelters in field plots	*Ditylenchus* spp., *Hemicycliophora* spp., *Hoplolaimus* spp., *Pratylenchus* spp., *Rotylenchus* spp., *Subanguina* spp.	Endoparasites responded positively to increased rainfall, ectoparasite abundance was unaltered	USA	Ankrom et al., 2020
Arid, semiarid and mesic grasslands	Precipitation manipulation by using rainout shelters in field plots	*Paratylenchus* spp., *Helicotylenchus* spp., *Hoplolaimus* spp., *Trichodorus* spp., *Xiphinema* spp., *Longidorus* spp.,	Increased precipitation in semiarid and mesic site increased and decreased root herbivory abundance, respectively.	USA	Ankrom et al., 2022

### 3.1 Impact of elevated temperature

With the present trend the global temperature is expected to exceed about 1.5-2°C warming in the coming decades (www.ipcc.ch). The winters in northern hemisphere will predictably be warmer allowing permafrost thawing to change the vegetation pattern (Ibelings et al., 2011; Van Hemert et al., 2015). Boland et al. (2004) reported that soybean cyst nematode *H. glycines*, after being identified in Canada in 1987, has spread to the northern and north-eastern provinces including Ontario, Essex, Kent, Lambton, Elgin, Perth, Haldimand-Norfolk, Middlesex, Glengarry, Prescott, Stormont, Huron and Oxford due to climate change (and also other factors). Since the 1960s, different species of *Meloidogyne* (*M. incognita*), *Pratylenchus* (*P. brachyurus*, *P. vulnus*), *Helicotylenchus* (*H. pseudorobustus*, *H. multicinctus*) and *Ditylenchus* (*D. dipsaci*) experienced a latitudinal shift towards northern hemisphere due to global warming (Bebber et al., 2013). On the contrary, a study with *H. schachtii* populations from Morocco, Spain, France, Germany, Austria, Poland and Ukraine indicated that global warming may not strongly affect the northern populations of the nematode species (Fournet et al., 2018). It is also predicted that an increase of 1°C in the mean temperature would cause northward expansion of virus transmitting longidorids and trichodorids by approximately 160-200 km in Great Britain (Neilson and Boag, 1996). The model showed that the populations of *X. diversicaudatum* (vector of strawberry latent ringspot and arabis mosaic viruses), *L. macrosoma* (vector of raspberry ringspot virus) and *L. attenuatus* (vector of tomato black ring virus) would migrate northwards to as far as northeast and southern Scotland. Colonization of newer geographical locations by virus transmitting PPNs will obviously pose a serious threat to the crop productivity. In India, climate change is influencing the geographic distribution pattern of rice root-knot nematode *M. graminicola*. Earlier considered to be a pest of upland rice only, *M. graminicola* has currently spread to all major rice growing belt in India including hill ecosystems and direct seeded rice (Dutta et al., 2012). In this line, it was observed that *M. graminicola* causes greater damage in rice roots under elevated CO_2_ conditions (700 ppm) than under ambient CO_2_ level (400 ppm) (Prasad and Somasekhar, 2009). Along with range shifting the rising temperature also causes differential changes in the abundance and distribution of the PPNs. A strong positive correlation between the incidence of *Longidorus caespiticola* and rise in temperature by 1°C was evidenced in the northern territories of United Kingdom (Boag and Neilson, 1996). Higher PPN abundance at increased temperature was also noticed in the Kashmir valley of India (Nisa et al., 2021). Although not conclusively proved, the reniform nematode *R. reniformis* was predicted to expand its distribution in USA due to global warming (Leach et al., 2009). Rising temperature also propelled the distribution of pine wilt nematode *B. xylophilus* and its vector beetle *Monochamus alternatus* in several parts of the world, where Asia, northern parts of Europe and America are particularly vulnerable (Hirata et al., 2017; Li et al., 2022). Earlier believed to be of temperate origin, pine wilt is now more prevalent in the warmer climates as high temperature and low precipitation in summer result in enhanced disease progression due to change in nematode distribution, vector incidence and water stress on pine trees (Rutherford et al., 1990). However, in Qinghai–Tibetan plateau rise in temperature caused reduction in the abundance of PPNs (Liu et al., 2022). Similarly, global warming (in combination with elevated CO_2_) decreased the PPN abundance in semiarid grasslands (Mueller et al., 2016). The elevated temperature will also influence the soil dwelling nematode communities and related decomposition pathways across the altitude gradients (Yu et al., 2021). A recent study with metabolic footprint based analysis predicted PPN abundance (and of fungivores and omnivores) to increase at the higher altitudes of Pir-Panjal mountain range and of Western Himalaya (Afzal et al., 2021). The host-seeking stages of some PPN species (*M. floridensis*, *M. incognita*, *D. phyllobius*, *R. reniformis*) prefer to migrate towards warmer soil when a temperature gradient is present (Dusenbery, 1988; Robinson, 1994; Leitao et al., 2021), but any direct correlation of such migration with global warming is scant. However, taking the lead from such studies, it can be assumed that global warming may result in higher accumulation of certain PPN species through horizontal and/or vertical migration in soil due to climate change. Nonetheless, in long run, how such migration will swing the PPN-crop interaction on geospatial leverage is a matter of speculation at the moment.

Apart from the latitudinal and longitudinal shifts, temperature rise will also alter the pest status of some nematode species. For example, elevated soil temperature reduces the population of *G. pallida*, but increases the population of *G. rostochiensis* in southern United Kingdom (Jones et al., 2017). Similarly, temperature rise is predicted to expand the range of *R. similis* (normally prevalent at lower altitudes) at higher altitudes of Sub-Saharan Africa, where *P. goodeyi* causes more damage at higher altitudes having a thermoregulated segregation (in review of Coyne et al., 2018). This will expose the banana and plantain grown at higher altitudes towards greater PPN damage. A similar investigation performed in Russia predicted the extensive spread of *G. rostochiensis* that has become an alarming invasive pest in Russia due to global warming (Pridannikov et al., 2022). Majority of the PPNs complete their life cycle within 2-4 weeks under favorable environment, and a slight change in temperature exerts considerable impact on their life cycle progression (Evans and Perry, 2009). Low temperature favors slower development, whereas faster development is seen under warmer conditions (Tyler, 1933; Tzortzakakis and Trudgill, 2005; Maleita et al., 2012). In this direction, rise in temperature owing to global warming results in multiple generations per season with shorter life cycle durations (Trudgill et al., 2005). Using simulated models, it was predicted that global warming would expand the distribution of potato cyst nematode *G. rostochiensis* and increase the number of generations per year by 2050 in Finland (Carter et al., 1996). An extensive pest risk analysis carried out by Ghini et al. (2008) showed that climate change would aggravate the damage potential of root-knot nematode *M. incognita* races 1, 2 and 4 in coffee plantations in Brazil with subsequent increase in number of generations per month during the future decades of 2020, 2050 and 2080. More number of nematode’s generations will definitely aid in increment of parasitic load to the crops with a negative impact on the production. On the other hand, high temperature has been found to cause sex reversal from female to male (which are non-parasitic in nature) in the parthenogenetic root-knot nematodes (Papadopoulou and Triantaphyllou, 1982). A recent study with Antarctic population of free-living nematode *Plectus murrayi* showed that rise in temperature increases the metabolic activities and O_2_ consumption thereby disrupting the ecosystem structure and function (Robinson et al., 2023). The authors, in context of carbon-limited soil ecosystem of polar habitat, speculated higher nematode abundance in deeper soil due to vertical range expansion. However, such increase in metabolism and O_2_ consumption can be expected in the soil inhabiting host-seeking PPN stages due to global warming, which might bring same effects (as above) or reduce their survival duration in soil due to high energy usage. These situations in a series indicate a dichotomy in plant-nematode parasitism under elevated temperature condition, where the climate change effects can be estimated by both increase and decrease in total parasitism. If so, more researches are needed to conclude the final outcome of the expected dualism at species-specific and location-appropriate levels.

Global warming has been found to cause tree mortality in the pine forests of Switzerland as high temperature favors the abundance of pine wilt nematodes (*B. mucronatus*, *B. xylophilus*), their vectors (bark beetle: *Tomicus minor*, *T. piniperda*), and stem fungi (another symbiotic partner), that negatively alters the host-tree resistance to incoming pathogens as they experience drought stress to a great extent (Rebetez and Dobbertin, 2004). Thus, high temperature is likely to exacerbate the symptoms of water stress in the host plants infected with PPNs and thereby affect their nutrition. Furthermore, climate change may affect the *R*-gene mediated resistance in plants against the root-knot nematodes because the resistance governed by *Mi* gene (cloned from wild tomato *Solanum peruvianum* and introgressed into cultivated species *S. lycopersicum*) is particularly lost at a temperature above 28°C (Williamson and Kumar, 2006). Although a number of heat stable *Mi* genes have been identified subsequently (El-Sappah et al., 2019), the efficacy of these genes in high heat stress is yet to be evaluated. A genome-wide study indicated that *Globodera* spp. respond to heat stress by expressing a number of heat tolerant genes, and *G. rostochiensis* was more thermotolerant than *G. pallida*. Molecular analyses revealed that the heat shock protein-coding gene (*hsp-110*) and its promoter were duplicated in *G. rostochiensis* genome (Jones et al., 2018). Similar acquisition of thermotolerant components in other nematode species during prolonged exposure to changing climate may give rise to more virulent and climate-adapted biotypes.

### 3.2 Impact of elevated carbon dioxide

It is generally accepted that elevated level of CO_2_ positively affects root-feeding nematodes as a result of proliferated root biomass (Hungate et al., 2000; Sticht et al., 2009; Hu et al., 2017; Wang et al., 2019). Studies under elevated (475 ppm) and ambient (360 ppm) CO_2_ levels in pasture land of New Zealand suggest that PPNs become more abundant at elevated CO_2_ conditions compared to other soil organisms (Yeates et al., 2003). The study particularly recorded excessive abundance of the PPN genera *Longidorous*, and assumed that the elevated level of CO_2_ had induced greater root biomass to bring positive effect on PPN incidence. In another study, different species of *Pratylenchus* were abundantly encountered (compared to other PPN species distribution) in the soil samples recovered from wetland or hydric soils collected from plots exposed to elevated CO_2_ (Yeates et al., 1999). Hoeksema et al. (2000) showed increased abundance of trichodorids under elevated CO_2_ in soils having low nitrogen content. The sensitivity of soil nematodes to CO_2_ enrichment under different cropping system shows nematode channel ratio (ratio of the number of bacterivorous and fungivorous nematodes in a given soil sample) to be considerably increased in sugar beet and wheat grown under elevated CO_2_ conditions in Germany (Sticht et al., 2009). Additionally, the PPN numbers were found to be significantly higher in both the crops at physiologically mature stage under elevated CO_2_. Nevertheless, total number of PPNs was higher in wheat than sugar beet, which may be a result of the variation in root system of the crops as well as the differences in composition of root-associated microflora or fauna under changing climate. A recent study found that, elevated CO_2_ level (500 ppm) causes canopy warming (+2°C) that significantly increases the rhizospheric PPN abundance, but decreases the total nematode diversity (Wang et al., 2021).

However, elevated CO_2_ conditions do not always positively regulate the PPN distribution and abundance. For example, Ayres et al. (2008) reported no change in total nematode abundance, species richness and species diversity index in the CO_2_ enriched soil (~350 ppm in excess of the ambient level) after conducting investigations at three different locations (Colorado and California in USA, Montpellier in France) in pasture plots. Similarly, Li et al. (2007; 2009) reported no alteration in the PPN abundance in rice-wheat rotation system under elevated CO_2_ condition in China, although elevated CO_2_ (enriched by 200 µmol CO_2_ mol^-1^ over 370 µmol CO_2_ mol^-1^ under free-air CO_2_ enrichment system) had increased the abundance of omnivores and predators, maturity index and structural index value of nematode assemblages at the jointing stage of wheat. They concluded that the interaction between residue incorporation or nitrogen fertilization and CO_2_ enrichment markedly influenced the soil nematode dominance and community indices. Eisenhauer et al. (2012) also recorded decrease in total PPN abundance under elevated CO_2_ condition in affecting the soil food web structures. However, Neher and Weicht (2013) observed decrease in abundance of the PPN genus *Aphelenchoides* under elevated CO_2_ condition in pine forests, whereas *Xiphinema* was unaffected and *Longidorus* was increased. Taken together the data from different ecosystems across the world, it can be concluded that elevated CO_2_ conditions either confer positive or negative or neutral impact on the PPN abundance and distribution.

Disease development in plants is dependent on three interacting factors, i.e. susceptibility of host, virulence of pest and pathogen, and conduciveness of environment (Agrios, 2005). The environmental factors being one of the indispensable components of the disease triangle are malleable to climate change, which ultimately determines the outcome of plant-PPN interaction in an epidemiological scale (Yeates and Newton, 2009). Although rising CO_2_ levels have differential (positive or negative or neutral) effect on PPN incidence, its effect on plant-PPN interaction is quite complex. For example, the PPN induced damage in grass species was comparatively greater in elevated CO_2_ condition than the same in ambient CO_2_ condition due to production of greater root biomass in the former case (Wilsey, 2001). On the contrary, PPNs responded neutrally to CO_2_ enrichment in grassland ecosystem despite infecting 3-32% greater root biomass of grass species in elevated CO_2_ condition than the same in ambient CO_2_ condition (Ayres et al., 2008). This idiosyncratic trend was correlated with decrease in root quality due to low root nitrogen content or increase in PPN antagonists’ population buildup. It is hypothesized that under elevated CO_2_ conditions, in order to maintain the same population level, PPNs may have to consume more plant material that contains low nitrogen content and soluble sugars, that ultimately leads to greater plant damage and reduced crop yield (Ayres et al., 2008). Climate change also influence the plant-PPN interaction by interfering with plant defense mechanism. Hamilton et al. (2001) predicted the performance of PPNs to alter under elevated CO_2_ by carbon-nutrient balance hypothesis, where increased CO_2_ facilitates photosynthesis and root growth on one hand, but also elevates the defensive secondary metabolites (phenols, terpenes, sesquiterpenes) as a result of higher accumulation of carbon in the plant tissue. In this line, Sun et al. (2011) observed elevated CO_2_ to repress the jasmonic acid (JA)-mediated defense signaling pathway in tomato when infected with root-knot nematode *M. incognita*. Likewise, increase in CO_2_ surged the root galling on JA defense-dominated genotypes, but no effect was seen in the JA defense-recessive or wild type genotypes. Furthermore, Cesarz et al. (2015) found that PPNs belonging to the ectoparasitic dorylaimid family Longidoridae do not benefit much (as indicated by no significant change in abundance compared to control) from the increased root biomass occurring under elevated CO_2_ conditions. The authors predicted this might be due to the activated plant defense elicited by more robust root system and alteration of soil carbon and nitrogen levels. Nevertheless, more focused research is needed in this area to draw any general conclusion about how the plant-feeding groups like dorylaimids, tylenchids and triplonchids respond to elevated CO_2_ level.

### 3.3 Impact of altered precipitation

Climate change affects the global precipitation by altering its amount, frequency and seasonal distribution (Tolle et al., 2013; Spinoni et al., 2014; Chen and Sun, 2017). The spatio-temporal variation of precipitation eventually influences the soil moisture that simultaneously impacts the PPN community as they heavily rely on water for activity, host finding, development and sustenance (Yeates et al., 1999). Based on a study at Tennessee, USA, Kardol et al. (2010) concluded that soil moisture most prominently impacts the PPNs than elevated temperature and CO_2_ under the threat of climate change. In general, the PPNs typically show an intense response to elevated rainfall than the nematodes of other trophic groups (Guogang et al., 2020). Under increased rainfall condition and greater long term mean annual precipitation, the PPNs derive more benefit due to concomitant boost in the plant biomass (Nielsen et al., 2014; Sylvain et al., 2014; Vandegehuchte et al., 2015; Olusanya et al., 2019). Similar observations were reported by Bristol et al. (2023), who found increase in PPN abundance in long-term due to elevated rainfall. The altered soil moisture level may also change the abundance and diversity of the soil inhabiting PPN community. For example, with increase in rainfall an increase in prevalence of the PPNs such as *M. minor*, *Heterodera* spp. and *Pratylenchus* spp. was reported from the grasslands and cereal cropping systems of Northern Ireland (Fleming et al., 2016). Such changes in PPN diversity and abundance may occur under climate change scenario, where it is plausible that a minor species may become a serious one due to temporal shift in precipitation. However, the effects of one year rainfall manipulation in New Jersey Pinelands of USA showed the PPN families Criconematidae, Hoplolaimidae and Pratylenchidae to be unaffected by precipitation manipulation, whereas *Aphelenchoides* sp. was negatively affected by droughts (Landesman et al., 2011). Similarly, Torode et al. (2016) found negligible changes in PPN abundance under altered precipitation. On the other hand, decreased precipitation frequency was found to increase the abundance of PPNs in lightly degraded grasslands *via* improvement of root biomass (Chen et al., 2021). But, under moderate and severely degraded conditions, the authors found the effect to be reversed through change in soil moisture and pH. The effects of changes in precipitation have also been studied in combination with the other climatic and agronomic factors. For example, climate warming and drought together decrease the plant production, rate of nutrient turnover and resource input (Kardol et al., 2010), which in turn may affect the PPNs. Sun et al. (2013) found combined application of water and nitrogen in soil to reduce the PPN (and other nematodes) abundance in organic horizon of temperate soil of China, whereas the opposite trend was seen in the mineral horizon. Based on the study, the authors opined the climate change driven alteration of precipitation and combined nitrogen application in soil may fatally disturb the soil nitrogen balance in due course of time. In this line, Ma et al. (2018) reported that the response of soil nematodes including the PPNs towards climate warming and drought depends upon the soil layers and growing seasons.

The climate change-driven changes in precipitation also affects the PPN parasitism in host plants. A study by Ankrom et al. (2020) reported that altered precipitation confers differential effect on PPN feeding strategies and their functional guilds. They found that the abundance of endoparasitic PPNs remained unaltered with increasing precipitation at the arid grassland site, increased at the semiarid site and declined at the mesic site. On the contrary, ectoparasitic PPN abundance was unaltered in the arid and semiarid site, but decreased in the mesic site. The variable response of ecto- and endoparasitic PPNs might have important implications in grassland productivity, because population pressure of PPNs aggravates the effect of climate change on host plants.It was suspected that elevated soil moisture level in the arid and semiarid conditions allowed active movement of the ectoparasites between root feeding sites thereby facilitating higher reproduction and completion of life cycle. However, increased top-down effect by predators in the mesic sites might decrease the abundance. Contrastingly, the negative response of endoparasites at the mesic site could be due to higher plant chemical defenses against initial invasion and establishment as increased soil water facilitates plant growth and defense, and the increased abundance at semiarid site could be due to positive response of nematodes to the increased precipitation. Later, Ankrom et al. (2022) analyzed the nematode sensitivity to climate change calculating different community analysis measures in grassland ecosystems at five different levels of manipulated precipitation over two year period. In arid ecosystem, under increased precipitation, nematode communities became enriched in species and a shift towards fungal feeder-dominated decomposition pathway was encountered. The semiarid ecosystem exhibited a decline in nematode maturity, structure and fungal feeder-dominated decomposition, although increased levels of species enrichment and PPN incidence were documented. Nematode communities in humid ecosystem (experiencing the enhanced precipitation) showed greater maturity and structure and switched to fungal decomposition channel, which reflected a decline in species enrichment and PPN incidence. Abundance of specific genera that were affected by enhanced precipitation level was *Eucephalobus* in the arid site and *Trichodorus* in the humid site. In this context, more PPN genera should be studied at cross-site levels to idealize their specific ecosystem responses to climate change.

The alteration in precipitation also disrupts the fragile balance between PPN communities and their natural parasitoids and predators (Chiew et al., 2011). The projected increase in the frequency of extreme dry years due to global warming lessens the predation pressure on PPNs thereby increasing their population pressure and plant infestation in the mesic grasslands of USA (Franco et al., 2019). The increased plant damage then worsens the drought effects by altering carbon cycling and ecosystem production. In continuation of the study, authors demonstrated in sub-humid grasslands that altered below-ground trophic interactions, under extreme drought, affect plant growth (due to change in carbon allocation for plants’ own physiological need) with reduced root biomass (Franco et al., 2020). Furthermore, Colagiero and Ciancio (2012) predicted that altered soil moisture regimes affect the PPN biocontrol agents in terms of their densities, emergence, development and spread. They predicted an increase in the spread of some moisture-dependent biocontrol agents such as *Catenaria* under high soil moisture content.

### 3.4 Impact of extreme climate events

Climate change intensifies the frequency of extreme weather events including heat waves, droughts, heavy downpours, wildfires, storms and hailstorms that seriously threatens the global food security and ecosystem services (Hasegawa et al., 2021). The nematodes, in general, possess a diverse array of structural and behavioral adaptations to survive in the climatic extremes or unpredictable stressed weathers (McSorley, 2003). For example, the *Meloidogyne* spp. can undergo sex reversal under a variety of stresses including nutritional deficiency, temperature fluctuation, increased host defense, reduced photosynthesis in the host to combat the adversities (McSorley, 2003). Additionally, PPNs can enter into cryptobiotic or anabiotic life-state of arrested development under drought events and regain normal body functions when the environment becomes favorable (Wharton, 1986). Wu et al. (2018) examined cryoprotective dehydration to enhance cold tolerance in *M. hapla* under freezing temperature. However, reports in relation to the other climatic extremes such as heat waves, wildfires, storms and their impacts on PPNs in the climate change continuum are extremely scant.

A study on Alpine meadow of Qinghai–Tibetan plateau showed the abundance of PPNs to significantly reduce due to excessive gradient warming with reduction in maturity index (Liu et al., 2022). Majdi et al. (2019) showed the broad range of temperatures (10, 15, 20, 25, 30 and 35°C) to alter population growth and body size of the free-living nematode genera *Plectus* and *Acrobeloides*; and predicted the heat waves to exert similar effects on the nematode communities under open field. Although not directly related, the results can be extrapolated onto the PPNs, where heat waves may affect their abundance and growth, but whether there will be any effect on the morphology or physiology is a matter of speculation. Further, heat waves have been found to negatively affect the plant’s photosynthetic ability (Chovancek et al., 2019), which may force the PPNs to undergo sex reversal. In addition, it may curb the plant’s defensive ability and post-infestation survival upon PPN attack.

While studying the effects of wildfires on soil-inhabiting microorganisms and mesofauna, Pressler et al. (2019) indicated the nematodes to be a weakly affected group in comparison to the bacteria, fungi and other microbes. However, within phylum Nematoda, response of different nematode groups towards wildfire events are uneven. In this direction, Bastow (2020) found maximum decrease in the abundance of PPNs than the bacterivorous and fungivorous nematodes. The author also observed that during the study period of four years no PPNs could revive, while the other groups recovered. This suggests that wildfires may lead to severe diminution of the PPN diversity and abundance in the soil. Recently, Renčo et al. (2022) found the post-fire recovery of the soil nematode communities to depend on the severity of the fire event. Other studies also revealed the PPNs to be the most detrimentally affected group amongst the soil nematodes during a wildfire event (Smolik and Rogers, 1976; Čerevková et al., 2013; Renčo and Čerevková, 2015; Abdoos and Saeedizadeh, 2017; Abdous and Saeedizadeh, 2017). However, all the studies have only concentrated on the abundance and recovery time of the PPNs post exposure to a fire event. In this context, future studies are necessary on the recovery mechanism(s) of the PPNs after fire, and identification of the factors behind their vulnerability and delayed resurgence as compared to other nematode groups.

The impact of severe storms (including cyclone, tornado, hurricane and typhoon) on the PPN community has been rarely studied. However, it can be predicted that strong winds can topple the plants thus contributing to reduce the vegetation stand, which may alter the associated rhizosphere-inhabiting ecto-, semiendo- and endoparasitic PPN abundance for a short-term. Nevertheless, in a long run it is expected that the PPNs will migrate towards the surrounding rhizospheres (having live roots) or rejuvenate the community structure as the plants will revive. Further, accidental introduction of a plant species in a new location by storm might re-shape the PPN diversity. Storms can also help in disseminating the PPNs in newer locations. For example, de Rooij-van der Goes et al. (1997) encountered a low number of PPN species in the drifted sand blown out during storm at the coastal foredunes of The Netherlands. These types of events can mostly be expected for the cyst nematodes (cysts containing viable eggs can blow out) or seed gall nematode (*Anguina* filled seeds may blow out), but the soil-inhabiting PPNs will largely remain unaffected.

## 4 PPN management under climate change scenario

Climate change effects are continually becoming visible in multifarious permutations. The events such as range expansion, wide distribution, increased generation, abundant reproduction, proliferated root biomass, lessened host defense are likely to aggravate the PPN colonization, abundance and damage. In this direction, the elevated temperature in winter months and temperate areas is likely to aid in higher abundance of some PPNs that will eventually impact the economic production in the respective regions. The expansion of range and invasion to newer areas may cause havoc due to availability of ‘no-hindrance’ for the nematodes, and as a result the consequent population build-up will be un-manageable. The situation maybe worsened with the increased reproduction rate and completion of above-normal generations per season. Frequent changes in humidity and precipitation profile may also favor some species over the others, and minor, occasional and seasonal pests are likely to achieve major pest status. Furthermore, the rise in temperature and UV radiation along with decrease in relative humidity may render several pest management strategies ineffective. Few events such as sex reversal, cryptobiotic life stages, reduced survival in open soil, altered pathogenicity may aid nullifying the atrocities. While assorting all the events in a line for a generalized conclusion, it seemingly creates a contraposition effect, where one set of factors are likely to aggravate the parasitic potential of the PPNs but the other set of factors seem to act in counter direction. Nevertheless, it is difficult to predict any overall common trend at present. Following the process of natural adaptation and evolution the PPNs will presumably respond according to their specific and regional distinctions. In this critical juncture we may no longer rely on the current PPN management tactics, and should better move to ‘climate-smart nematode management’ aiming reduced crop loss and enhanced ecosystem services with minimal GHG emission and emphasized resilience of agricultural systems (Heeb et al., 2019; www.fao.org/3/i1881e/i1881e00.pdf).

Integrated nematode management (INM) practices involve the combination of different control measures such as cultural, physical, botanical, biological and chemical in order to suppress the population level of PPNs below the economic threshold. Accordingly, the PPN management strategies must be tweaked in such a way that it should suit the impending environmental fluctuations. For example, flooding and fallowing are widely adopted cultural tactics for PPN management (Rhoades, 1982; Bridge, 1996; Sikora et al., 2005; Chen and Tsay, 2006). However, flooding amplifies the GHG emission by disrupting the C and nutrient cycling (Sánchez-Rodríguez et al., 2019); and fallowing increases N_2_O emission when no nitrogenous fertilizer is applied in soil (Mosier et al., 1991; Lemke et al., 1999). In this context, it is advisable to introduce any PPN-antagonistic cover crop (e.g. grain sorghum, millets, oil radish, Mulato grass, sorghum-sudangrass) in the cropping system that potentially benefits the environmental quality under different cropping systems (Grant et al., 2002; Asmus et al., 2008; Liebig et al., 2010; Mateille et al., 2020; Oram et al., 2020; Khanal and Harshman, 2022). As alterations in the summer and winter month durations divert the PPN population dynamics in soil, few adjustments in the cropping schedule and associated crop management practices would be necessary to avert the damage in crops. The sustainable management tactics such as biofumigation and crop rotation have also assumed greater significance under changing climate scenario for improving the carbon sequestration in agroecosystems (Somasekhar and Prasad, 2012). Further, it has been observed that incorporation of organic green manures and soil solarization with addition of animal manures contributes to GHG emission (Rochette et al., 2004; Toma et al., 2019; Lee et al., 2021). Thus, use of farmyard manure (FYM) seems relatively safer with comparatively lower GHG production (Mori, 2018).

It is also plausible that global warming will impact the activity of the nematode antagonistic bacterial and fungal bioagents, but it is an underexplored territory so far. Exposing the *M. arenaria* second-stage juveniles (J2s) to 35-40°C or incubating the soil-dwelling *Pasteuria penetrans* (*Meloidogyne* antagonistic obligate bacterium) endospores at 50°C and above resulted in jeopardized attachment of bacterial endospores to the nematode cuticle (Freitas et al., 1997). In another study, Luambano et al. (2015) found the number of *Pochonia chlamydosporia* propagules to increase with increasing soil temperature resulting in elevated egg parasitism. Similarly, the nematicidal fungus *Metarrhizium anisopliae* was found in abundance with cover crop growth under enriched CO_2_ condition (Rezácová et al., 2005; Karabörklü et al., 2022). Additionally, the altered climatic factors are supposed to bring mismatch between the PPNs and their antagonists in time and space as both are exposed to open environment during interaction. In view of this, rise in temperature, UV radiation (due to increased thinning of ozone layer) and decreased relative humidity due to climate change may alter their hyperparasitic potential under field conditions. However, despite obvious relevance it is difficult to predict any trend for alteration of total soil suppressiveness or biocontrol efficiency in response to climate change where such antagonistic reactions (as employed by biocontrol agents) are part of naturally coevolving host-parasite interactions (Preston et al., 2003; Rafaluk-Mohr et al., 2018).

The adverse effects are also correlated with the host physiology changes especially with *R*-gene mediated resistance to PPNs deployed *via* conventional or molecular breeding (Sun et al., 2011). The temperature-modulated expression of the *R*-genes, along with the crosstalks between defense-signaling pathways and climate change induced stresses will possibly alter plant resistance potential. The general plant immune responses as governed by histone modifications, activated protein kinase cascades, reactive oxygen species (ROS) development and fluctuations in membrane fluidity are highly responsive to temperature sensing (Venkatesh and Kang, 2019). In this line, the global warming is expected to alter the general commute of the underlying pathways thereby affecting the defense against PPNs. Such changes in host resistance, in accordance to natural selection pressure, may also bring forth new PPN biotypes (more virulent or resistance breaking) with altered phenology, behavior and parasitic potential. Though emergence of new biotypes due to climate change was encountered in few insect species (Ramos et al., 2018; Kumar et al., 2021), this type of instances is largely unknown in any PPN to date. However, essential studies need to be conducted for enhanced crop sustainability in the PPN resistance – climate change continuum, where novel biotechnological approaches can offer a promising solution (Fuller et al., 2008).

Temperature fluctuations along with erratic precipitation due to climate change can potentially affect the persistence and availability of chemical nematicides in soil, which would ultimately impact the PPN control measures (Delcour et al., 2015). The demand for nematicides among farming communities is likely to be higher for the expected greater incidence of PPNs under changing climates. At present only a handful of nematicides (e.g. fluensulfone, fluopyram, fluazaindolizine, tioxazafen, ethanedinitrile, spirotetramat) are available in the global market (Desaeger et al., 2020), but their wide-scale usage is limited for all types of crops because of label claim restrictions. Information must be generated on the fate of nematicides in response to climate change. Although the approach may prove efficient in curbing the PPN problem in short-term, but not advisable for long-term solutions. Following the worldwide aim to reduce pesticide usage in the current era (Gatto et al., 2016; Jacquet et al., 2022), we cannot recommend any escalated nematicide use in the future. We must come up with a solution at the earliest how best the INM tools can be used in a sustainable way and this must be a major thrust for research at present.

Holistically, we require adaptive management strategies to combat and cope with the altered situation. Undoubtedly, the changed pest status will require extra efforts to mitigate such ‘hidden enemies’ of crop production. The changes in PPN species diversity and abundance will expectedly lead to jeopardy in effectiveness of the existing pest monitoring systems. Hence, new and sophisticated tools should be designed or existing methods should be improvised to assess the distribution dynamics and seasonal phenology of the PPNs. Also, scenario planning is necessary considering the non-climatic factors function hand-in-hand with climate change variables. A thorough analysis inclusive of PPN species and biotypes at spatiotemporal scale for the apprehended risk and adaptation is needed for the economically important PPNs at regional basis. Improved risk assessment and potential intensification of pest endurance tactics with wider inter- and intra-center collaborations will help developing new integrated pest management (IPM) modules and their disseminations. Use of tools such as host plant resistance, biopesticides, plant-based products, nurturing the natural enemies will surely contribute in the crisis as viable alternatives. A win-win state can be achieved for sustainable PPN management under changing climate with priority towards primary production, ecosystem services, and socio-economic feasibility of the farming practices.

## Author contributions

TD Conceived, formulated and edited the Manuscript. VP formulated the Manuscript. All authors contributed to the article and approved the submitted version.
